# Supplementary material for: TRIM40 is a pathogenic driver of inflammatory bowel disease subverting intestinal barrier integrity
Source: Nat Commun. 2023 Feb 9;14:700. doi: 10.1038/s41467-023-36424-0 (PMC9908899; doi:10.1038/s41467-023-36424-0)
Supplement: Supplementary file 1 — Supplementary Information [file 41467_2023_36424_MOESM1_ESM.pdf]

TRIM40 is a pathogenic driver of inflammatory bowel disease subverting intestinal barrier integrity

Sujin Kang[1], Jaekyung Kim[1], Areum Park[1], Minsoo Koh[1], Wonji Shin[1], Gayoung Park[1], Taeyun A. Lee[1], Hyung Jin Kim[1], Heonjong Han[1,2], Yongbo Kim[2], Myung Kyung Choi[1], Jae Hyung Park[3], Eunhye Lee[1], Hyun-Soo Cho[1], Hyun Woo Park[3], Jae Hee Cheon[4,5], Sungwook Lee[2], Boyoun Park[1]

Supplementary Figures

Supplementary Figures
Supplementary Figure legends

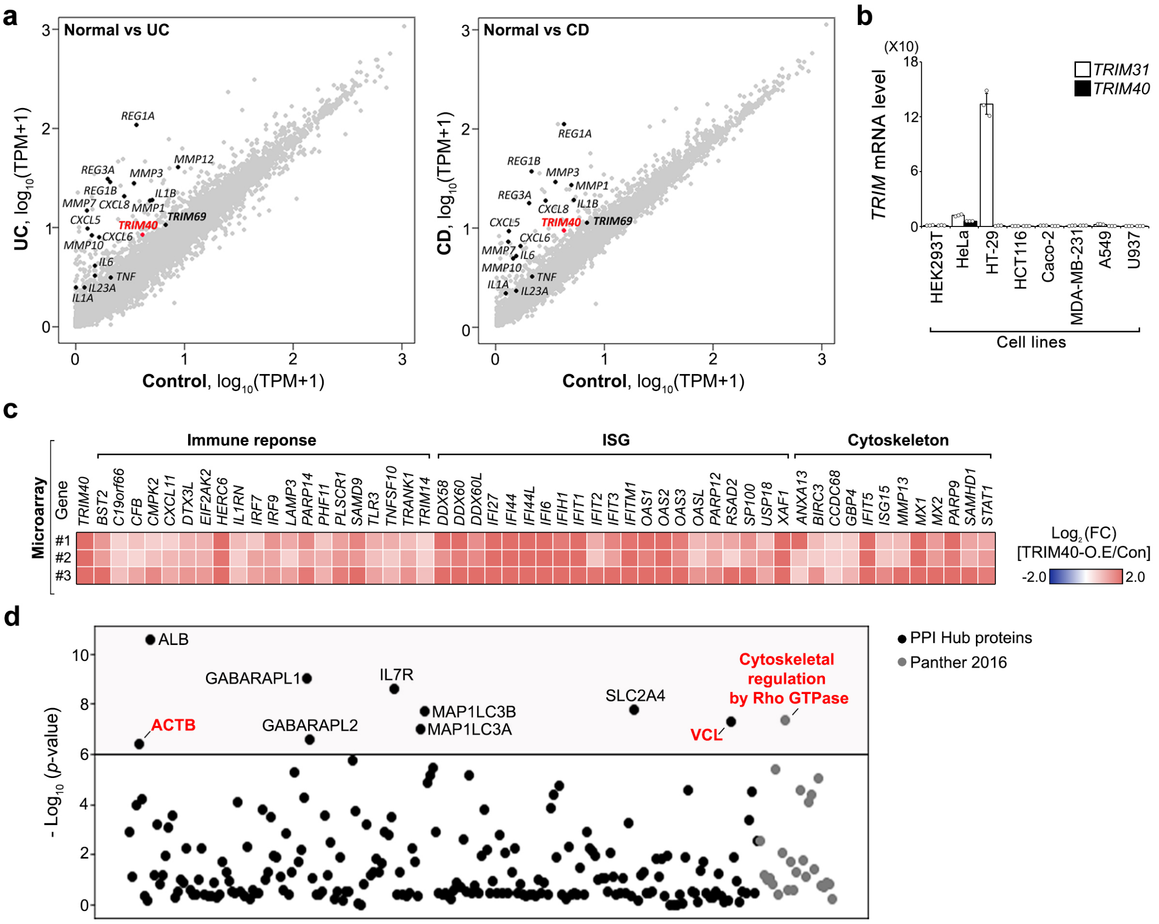

Supplementary Fig. 1. Aberrant TRIM40 upregulation is linked to IBD. **a**, Scatter plots representing the highly inducible signature genes associated with UC or CD (fold-change [FC] > 2) compared to healthy individuals (GSE117993). **b**, qPCR showing relative mRNA expression of *TRIM40* and *TRIM31* in various human cell lines (HEK293T [embryonic kidney], HeLa [cervix], HT-29 [colon], HCT116 [colon], Caco-2 [colon], MDA-MB-231 [breast], A549 [lung], and U937 [lymphocyte]). Data are representative of three independent experiments. **c**, Heat map representing relative log_2 values for fold-change of differentially up-regulated genes measured by microarray in HT-29 cells stably expressing Myc-TRIM40 compared with control. Red or blue colors represent high or low fold-change, respectively. Microarray results were obtained in three independent experiments (#1~#3). **d**, Manhattan plots showing analysis and classification of TRIM40-interacting proteins. Co-immunoprecipitated proteins with anti-Myc antibody in HT-29 cells expressing control vector or Myc-TRIM40 were separated and visualized by SDS-PAGE and silver staining, and subsequently analyzed by liquid chromatography with tandem mass spectrometry (LC-MS/MS). Data were analyzed by using protein-protein interaction (PPI) Hub proteins and Panther 2016 database. Black or gray circles represent PPI Hub proteins or Panther 2016 results, respectively. Red-letters indicate the cytoskeleton regulation-related proteins. *P* values are determined by fisher's exact test.

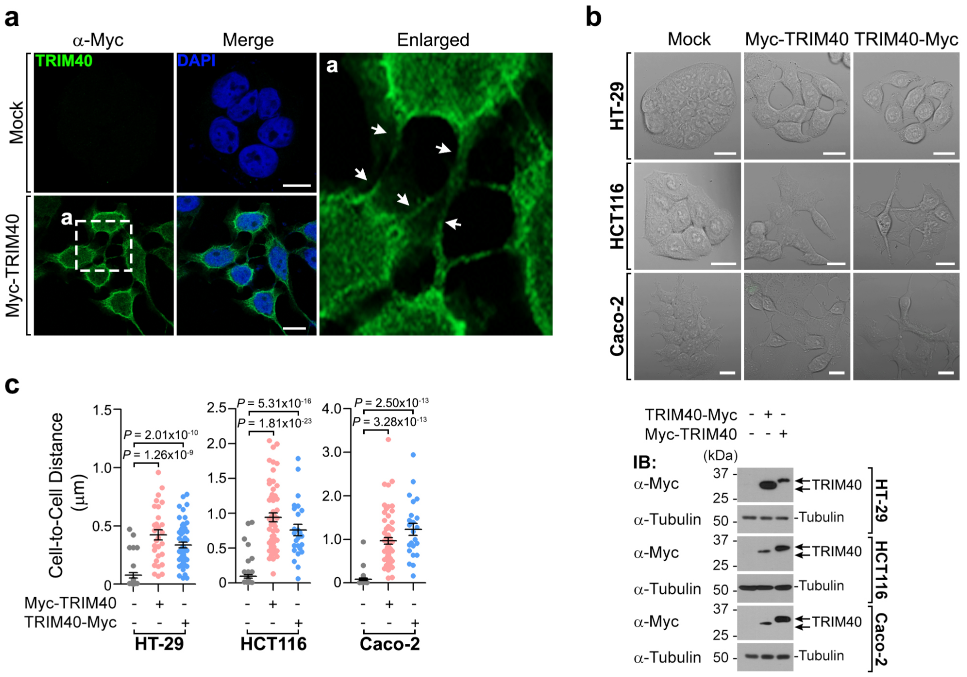

Supplementary Fig. 2. TRIM40 overexpression disrupts cell-to-cell contacts. **a**, Confocal microscopic analysis of TRIM40 (green) in HT-29 cells expressing control vector and Myc-TRIM40. Nuclei were stained with DAPI (blue). Enlarged views of the regions denoted by the white dashed squares. White arrows represent filamentous stretches of TRIM40. Scale bars, 10 µm. **b**, Differential interference contrast images showing morphological deformation in HT-29, HCT116, and Caco-2 cells expressing Myc-TRIM40 or TRIM40-Myc compared with control. Scale bars, 10 µm. The degree of TRIM40 overexpression was analyzed by immunoblot (bottom). Tubulin was used as a loading control. **c**, Graphs showing comparison of cell-to-cell distance in different epithelial cell lines, HT-29, HCT116, or Caco-2 cells, stably expressing Myc-TRIM40 or control vector. HT-29, control (n = 33), Myc-TRIM40 (n = 29), and TRIM40-Myc (n = 55); HCT116, control (n = 58), Myc-TRIM40 (n = 56), and TRIM40-Myc (n = 25); Caco-2, control (n = 34), Myc-TRIM40 (n = 65), and TRIM40-Myc (n = 23). Distances were analyzed by ImageJ software (V1.8.0). P values are determined by unpaired two-tailed t test. (n, biologically independent measurements of the distance between cells; mean ± SD). All Data are representative of three independent experiments and source data are provided as a Source Data file.

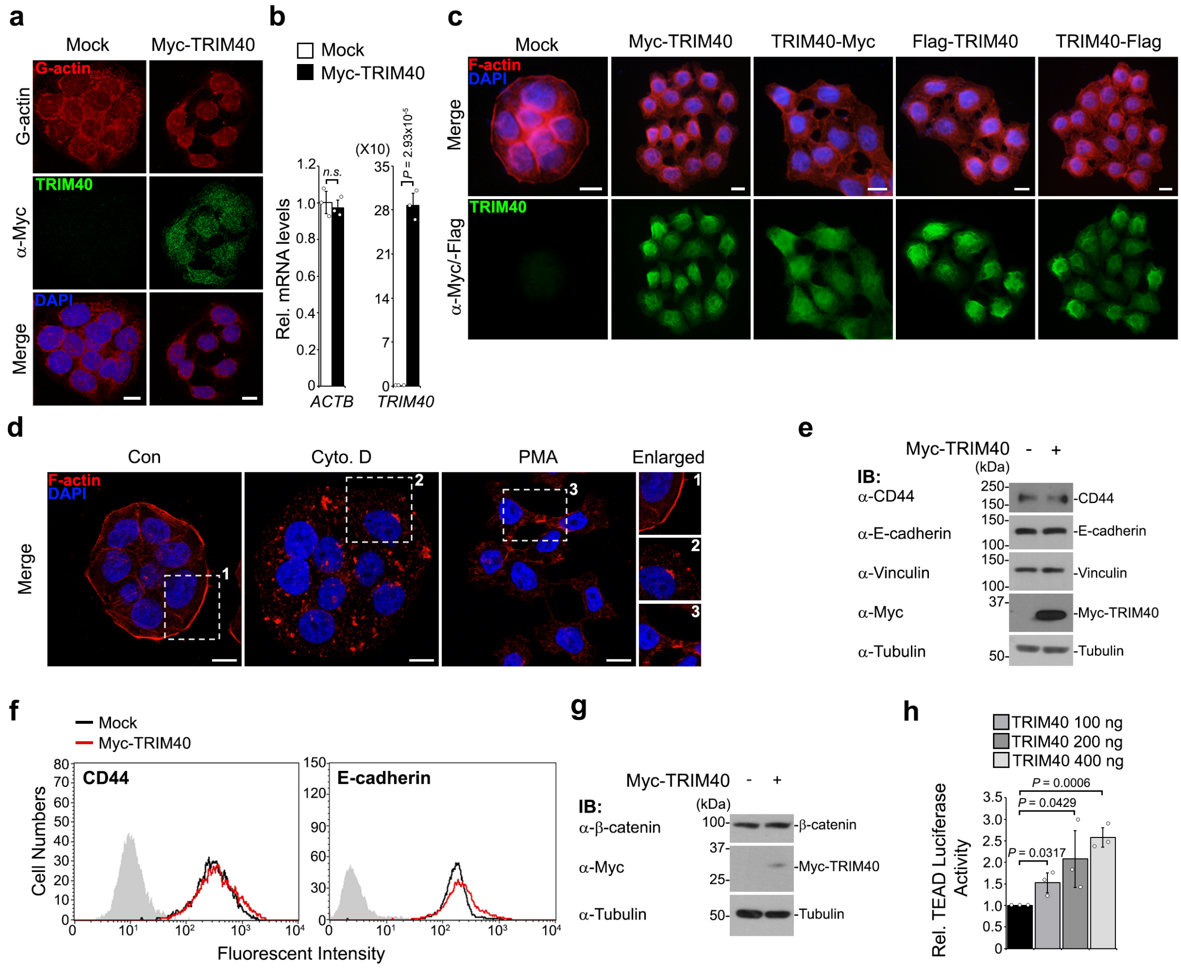

Supplementary Fig. 3. TRIM40 overexpression affects cortical actin stability. **a**, Confocal microscopic analysis of G-actin distribution in HT-29 cells expressing control vector and Myc-TRIM40. Cells were stained with DNase I (G-actin, red) or anti-Myc antibodies (TRIM40, green), and nuclei were stained with DAPI (blue). Scale bars, 10 μm. **b**, qPCR showing relative mRNA levels of *ACTB* (β-actin) or *TRIM40* in HT-29 cells expressing control vector and Myc-TRIM40. *n.s.*; not significant. **c**, Immunofluorescence assay of F-actin in HT-29 cells expressing Myc- or Flag-tagged at either N- or C-terminus of TRIM40. Nuclei were stained with DAPI (blue). Scale bars, 10 μm. **d**, Confocal microscopic analysis of F-actin in HT-29 cells after treatment with 0.25 μM cytochalasin D (Cyto. D) or 100 ng/ml phorbol 12-myristate 13-acetate (PMA) for 2h or 4 h, respectively. Cells were stained with phalloidin (F-actin, red) and nuclei were stained with DAPI (blue). Scale bars, 10 μm. **e**, Immunoblot showing protein expression levels of CD44, E-cadherin, or vinculin in HT-29 cells expressing control vector and Myc-TRIM40. Cell lysates were immunoblotted with indicated antibodies. Tubulin was used as a loading control. **f**, The cell surface expression level of junctional proteins, CD44 and E-cadherin, in HT-29 cells expressing control vector and Myc-TRIM40. Cell surface expression levels of CD44 and E-cadherin were assessed by flow cytometry analysis. Forward (FSC) and side scatter (SSC) were used for gating of mononuclear cells to exclude non-viable cells. **g**, β-

catenin protein levels in HT-29 cells expressing control vector and Myc-TRIM40. Cell lysates were immunoblotted with the indicated antibodies. Tubulin was used as a loading control. **h**, Graph representing an increase in TEAD activity by TRIM40 overexpression. Transcriptional activity of TEAD measured by luciferase assay with 8 × GTIIC-reporter plasmid containing tandem TEAD-binding sites in 293A cells transiently expressing different concentrations of TRIM40 plasmid. *P* values are determined by unpaired two-tailed *t* test in **b** and **h**. (n = 3 biological independent experiments, mean ± SD) All Data are representative of three independent experiments and source data are provided as a Source Data file.

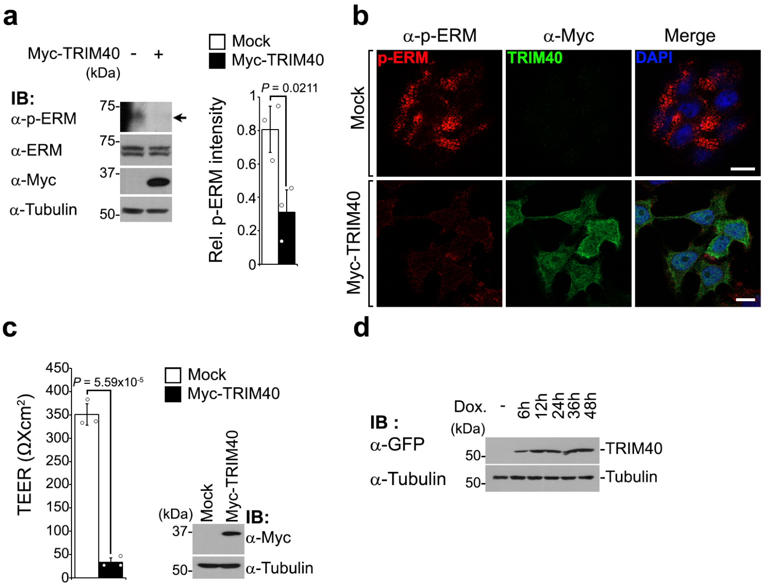

Supplementary Fig. 4. TRIM40 inhibits ERM phosphorylation**.** **a**, Immunoblots showing decreased phosphorylated ERM (p-ERM) levels in HT-29 cells expressing Myc-TRIM40 compared with the control vector. Quantification of the band intensity of p-ERM (right graph). Tubulin was used as a loading control. **b**, confocal fluorescent images of Myc-TRIM40 (green) and p-ERM (red) in HT-29 cells expressing control vector or Myc-TRIM40. Nuclei were stained with DAPI (blue). Scale bars, 10 µm. **c**, Transepithelial electrical resistance (TEER) values of Caco-2 cells expressing control vector or Myc-TRIM40 grown on transwell inserts. Myc-TRIM40 overexpression in Caco-2 cells was confirmed by immunoblot analysis (right blots). Tubulin was used as a loading control. **d**, Dox-inducible GFP-TRIM40 overexpression in HT-29 cells was confirmed by immunoblot analysis. Tubulin was used as a loading control. *P* values are determined by unpaired two-tailed *t* test in **a** and **c**. (*n* = 3 biologically independent experiments, mean ± SD) Data are representative of three independent experiments and source data are provided as a Source Data file.

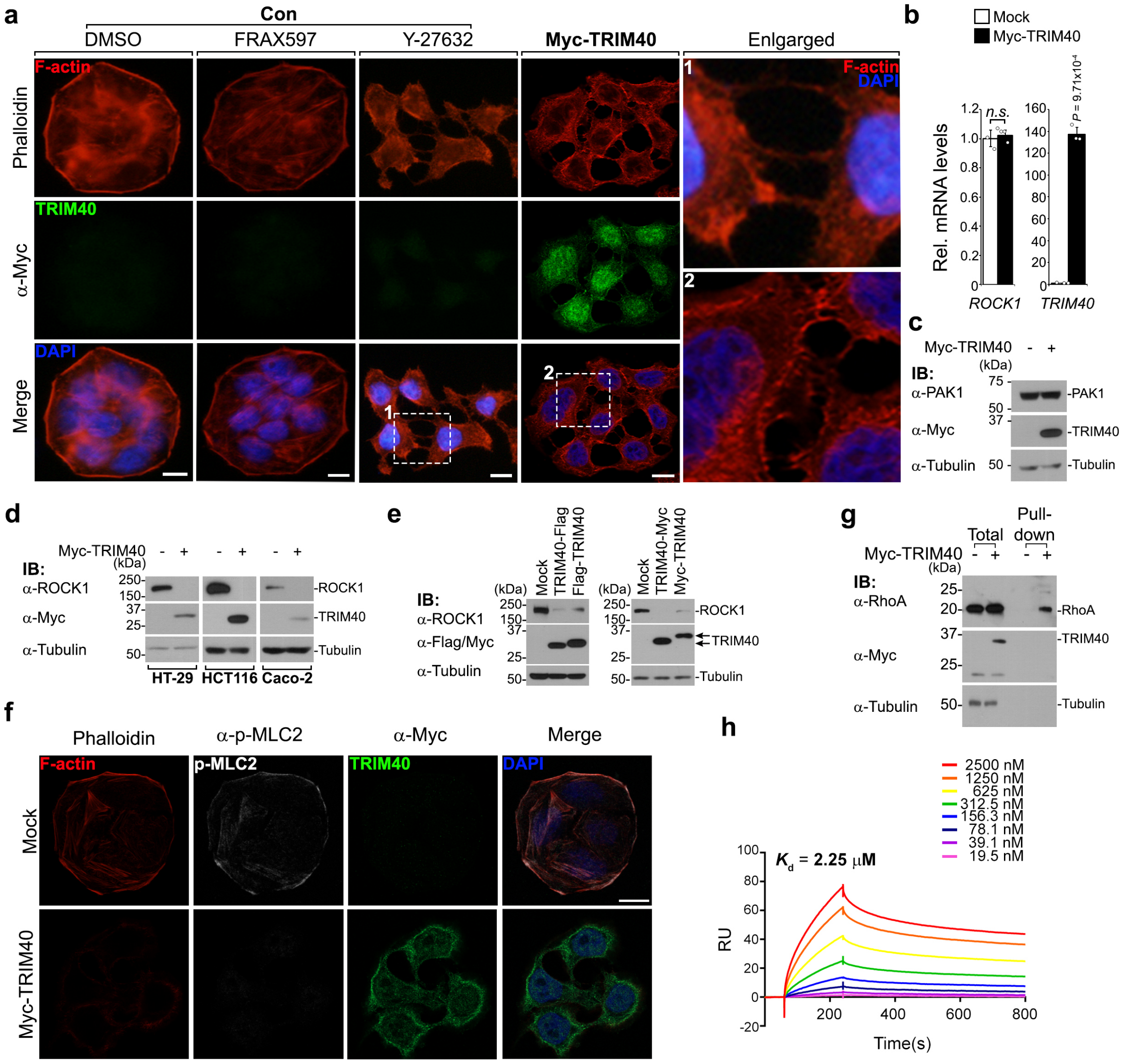

Supplementary Fig. 5. TRIM40 regulates RhoA-ROCK1 signaling by reducing ROCK1 protein levels. **a**, Immunofluorescence assay of F-actin distribution after inhibiting PAK1 or ROCK1. HT-29 cells were treated with PAK inhibitor (FRAX597, 5 μM) or ROCK1 inhibitor (Y-27632, 10 μM) for 4 h or 6 h, respectively. F-actin (red) and TRIM40 (green) were stained with phalloidin and anti-Myc antibodies, respectively. Nuclei were stained with DAPI (blue). Scale bars, 10 μm. Enlarged views of the regions denoted by the white dashed squares. **b**, qPCR showing relative mRNA levels of *ROCK1* and *TRIM40* in HT-29 cells stably expressing control vector or Myc-TRIM40. *P* values are determined by unpaired two-paired *t* test. (n = 3 biological independent experiments, mean ± SD) *n.s.*; not significant. **c**, PAK1 proteins levels in HT-29 cells stably expressing control vector or Myc-TRIM40. Cells were lysed and immunoblotted with the indicated antibodies. **d**, Evaluating ROCK1 protein levels in HT-29, HCT116, and Caco-2 cells expressing control vector or Myc-TRIM40. Cells were lysed and

then immunoblotted with the indicated antibodies. **e**, ROCK1 proteins levels in HT-29 cells expressing control vector, Myc- or Flag-tagged at either N- or C-terminus of TRIM40. Cells were lysed and then immunoblotted with the indicated antibodies. Tubulin was used as a loading control in c, d, e. **f**, Confocal microscopic analysis of F-actin and phosphorylated MLC2 (p-MLC2) in HT-29 cells expressing control vector or Myc-TRIM40. F-actin was stained with phalloidin (F-actin, red). p-MLC2 (white) and TRIM40 (green) were stained with anti-p-MLC2 and anti-Myc antibodies, respectively. Nuclei were stained with DAPI (blue). Scale bars, 10 µm. **g**, Rhotekin RBD (Rho-binding domain) pull down assay from HT-29 cells stably expressing control vector or Myc-TRIM40. The cell lysates were immunoprecipitated with glutathione resin, and the purified proteins were immunoblotted with the indicated antibodies. Tubulin was used as a loading control. **h**, Surface plasmon resonance (SPR) assay of the ROCK1 kinase domain (1~415 amino acids) concentration dependent binding to the TRIM40, representing a direct interaction of TRIM40 with ROCK1 (K_d = 2.25 µM). Data are representative of three independent experiments and source data are provided as a Source Data file.

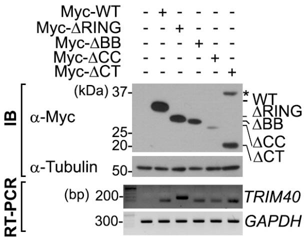

Supplementary Fig. 6. Protein and mRNA expression levels of wild-type TRIM40 and its deletion mutants. RT-PCR and immunoblot showing mRNA or protein expression levels of WT-TRIM40 or TRIM40 deletion mutants in HT-29 cells. HT-29 cells expressing control vector, WT-TRIM40, or TRIM40 deletion mutants were lysed and then immunoblotted with the indicated antibodies. PCR was performed with cDNA and the indicated primers. *; dimeric forms of TRIM40ΔCT. Tubulin or *GAPDH* was used as a loading control. Data are representative of three independent experiments and source data are provided as a Source Data file.

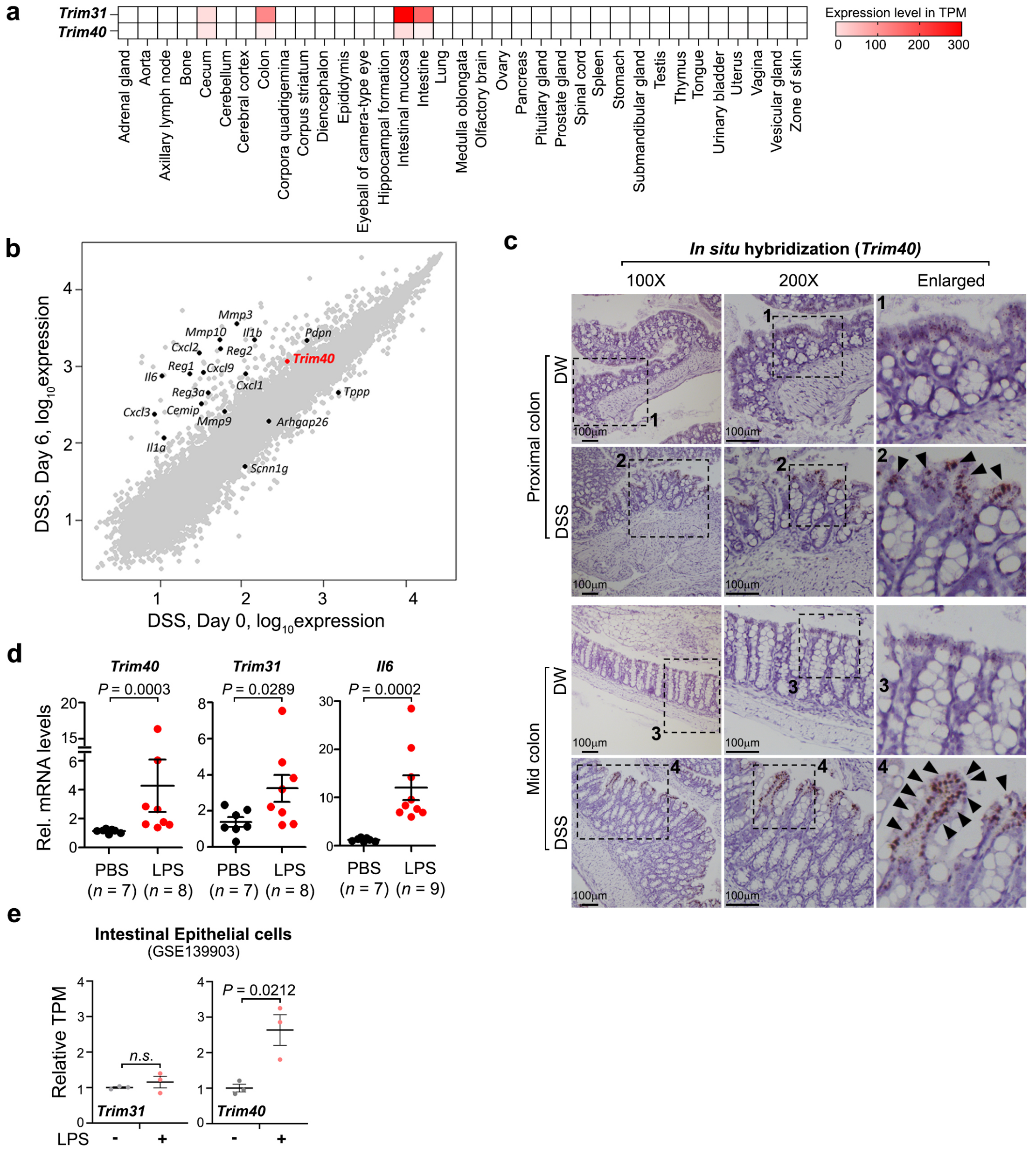

Supplementary Fig. 7. *Trim40* mRNA expression levels are increased in the DSS- or LPS-induced inflammation. **a**, The gene expression level of *Trim40* and *Trim31* in mouse tissues from RNA-seq datasets of FANTOM5 project. **b**, Microarray analysis showing increased expression of *Trim40* in colon tissues from DSS-treated mice compared with control (GSE22307). **c**, RNAscope *in situ* hybridization (ISH) showing *Trim40* mRNA expression in the proximal and mid colon tissues from WT male mice after 2% DSS or DW administration for 7 days. Brown staining (black arrow heads) indicates positive *Trim40* mRNA. Black dashed squares are shown the enlarged region (#1-4). Scale bars, 100 µm. **d**, qPCR showing relative mRNA levels of *Trim40*, *Trim31*, and *Il6* in distal colon from male mice intraperitoneally challenged with LPS (10 mg/kg body weight) (PBS group, *n* = 7; LPS group, *n* = 8 to 9). *P* values are determined by unpaired two-tailed *t* test (*n*, numbers of mice; mean ± SD). **e**, The

gene expression level of *Trim40* and *Trim31* in RNA-seq dataset from intestinal epithelial cells of mice treated with PBS or LPS (GSE139903). *P* values are determined by unpaired two-tailed *t* test (*n*, numbers of samples; mean ± SEM) Data are representative of three independent experiments and source data are provided as a Source Data file.

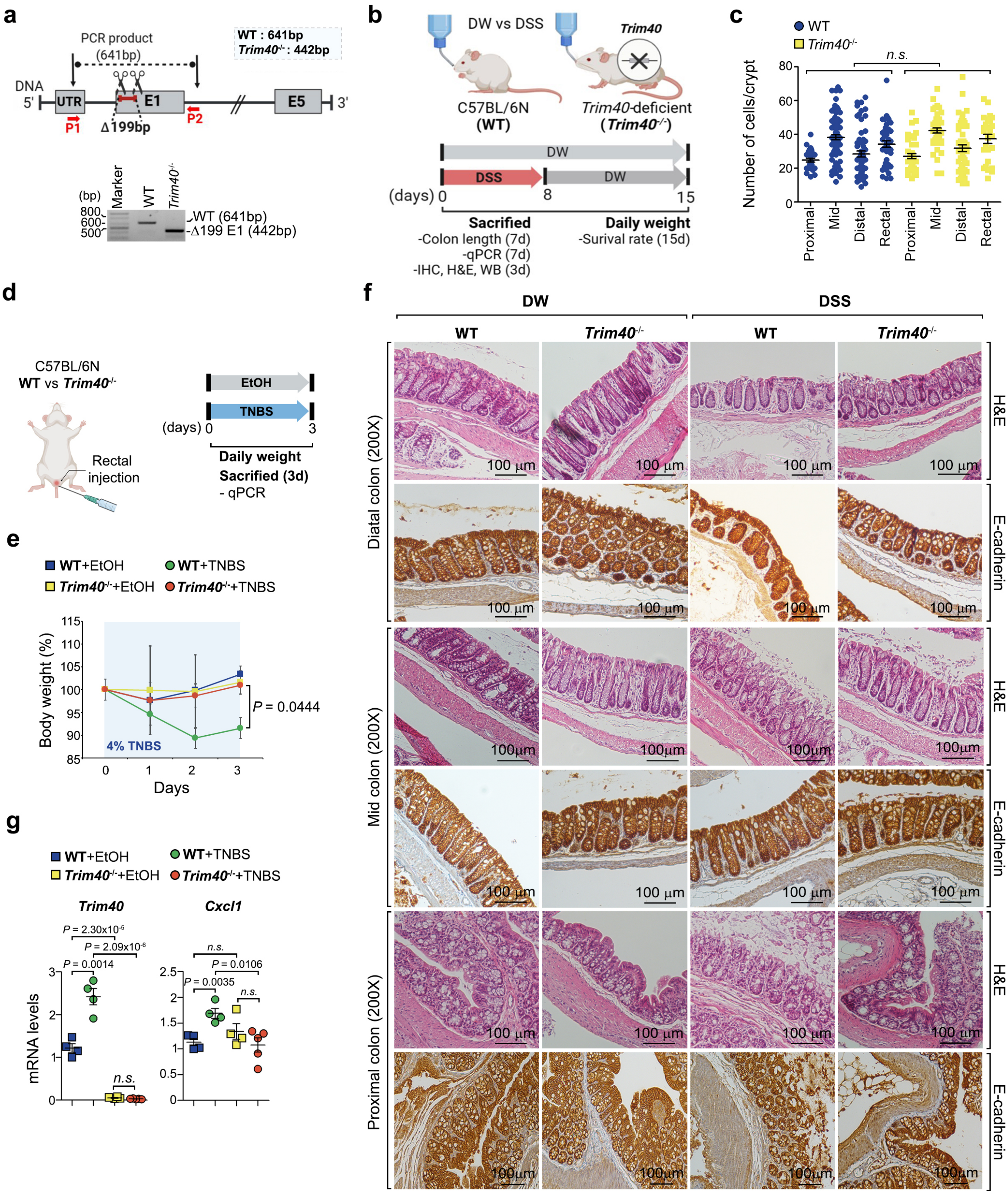

Supplementary Fig. 8. *Trim40*-dificient mice are protective to chemically induced colitis. **a**, A schematic representation showing CRISPR/Cas9-mediated gene-targeting strategy to generate *Trim40*$^{-/-}$ male mice. E; exon, P1/P2 and red arrows; PCR primers recognizing 5'-UTR or Intron 1 positioned in *Trim40*. Bottom gel, PCR products amplified from the targeted region of genomic DNA revealed genotypes of mice. **b**, Experimental design of DSS-induced colitis in WT and *Trim40*$^{-/-}$ male mice (image created with BioRender.com). **c**, Graph showing comparison of cell numbers in crypt from distal, mid, proximal, and rectal colon between WT (Proximal, *n* = 28; Mid, *n* = 71; Distal, *n* = 61; Rectal, *n* = 42) and *Trim40*$^{-/-}$ (Proximal, *n* = 40;

Mid, *n* = 46; Distal, *n* = 52; Rectal, *n* = 26) mice. *P* values are determined by unpaired two-tailed *t* test. (*n*, numbers of cells, mean ± SD) *n.s.*; not significant. **d**, Experimental design of TNBS-induced colitis in WT and *Trim40*$^{-/-}$ male mice (image created with BioRender.com). **e**, Relative loss of body weight in male mice for 3 days after 4% TNBS rectal injection. WT mice (EtOH group, *n* = 4; TNBS group, *n* = 4), *Trim40*$^{-/-}$ mice (EtOH group, *n* = 4; TNBS group, *n* = 5). *P* values are determined by unpaired two-tailed *t* test. (*n*, numbers of mice, mean ± SEM) **f**, Representative H&E staining and IHC staining of distal, mid, and proximal colon at day 3 after DSS exposure. WT and *Trim40*$^{-/-}$ male mice were administrated to 1% DSS in drinking water for 3 days and euthanized on day 3. Histological changes and difference between WT and *Trim40*$^{-/-}$ mice were examined by H&E staining and IHC for E-cadherin. (*n* ≥ 3 biological independent experiments) **g**, qPCR showing relative mRNA levels of *Trim40* or *Cxcl1* in 4% TNBS-induced colitis for 3 days. The expressions of *Trim40* or *Cxcl1* were analyzed in the distal colon tissue from WT male mice (EtOH group, *n* = 4; TNBS group, *n* = 4) and *Trim40*$^{-/-}$ male mice (EtOH group, *n* = 4; TNBS group, *n* = 5). *P* values are determined by unpaired two-tailed *t* test. (*n*, numbers of mice, mean ± SD) *n.s.*; not significant. Source data are provided as a Source Data file.
