# Supplementary material for: TRIM40 is a pathogenic driver of inflammatory bowel disease subverting intestinal barrier integrity
Source: Nat Commun. 2023 Feb 9;14:700. doi: 10.1038/s41467-023-36424-0 (PMC9908899; doi:10.1038/s41467-023-36424-0)
Supplement: Supplementary file 3 — Description of Additional Supplementary Files [file 41467_2023_36424_MOESM3_ESM.pdf]

Description of Additional Supplementary Files

File Name**: Supplementary Data 1**

Description: Substantial changes in genes that are signatures of IBD, including cytoskeleton-related and interferon (IFN)-stimulating genes (ISGs) in TRIM40-overexpressing HT-29 cells. Overlap in genes significant analyzed by two RNA-seq and three microarray experiments are listed in sheet #1 and #2, respectively. Classification of TRIM40-interacting proteins is listed in sheet #3.

File Name**: Supplementary Data 2**

Description: Reagents and antibodies (sheet #1), cloning primer sequences (sheet #2), and primer sequences of RT-PCR, qRT-PCR, or mouse genotyping (sheet #3) used in this study.
