# Supplementary material for: TRIM40 is a pathogenic driver of inflammatory bowel disease subverting intestinal barrier integrity
Source: Nat Commun. 2023 Feb 9;14:700. doi: 10.1038/s41467-023-36424-0 (PMC9908899; doi:10.1038/s41467-023-36424-0)
Supplement: Supplementary file 6 — Reporting Summary [file 41467_2023_36424_MOESM6_ESM.pdf]

nature portfolio

Corresponding author(s): Jae Hee Cheon, Sungwook Lee, Boyoun Park

Last updated by author(s): Jan 2, 2023

Reporting Summary

Nature Portfolio wishes to improve the reproducibility of the work that we publish. This form provides structure for consistency and transparency in reporting. For further information on Nature Portfolio policies, see our Editorial Policies and the Editorial Policy Checklist.

Statistics

For all statistical analyses, confirm that the following items are present in the figure legend, table legend, main text, or Methods section.

n/a	Confirmed	
☐	☒	The exact sample size (*n*) for each experimental group/condition, given as a discrete number and unit of measurement
☐	☒	A statement on whether measurements were taken from distinct samples or whether the same sample was measured repeatedly
☐	☒	The statistical test(s) used AND whether they are one- or two-sided<br>*Only common tests should be described solely by name; describe more complex techniques in the Methods section.*
☒	☐	A description of all covariates tested
☐	☒	A description of any assumptions or corrections, such as tests of normality and adjustment for multiple comparisons
☐	☒	A full description of the statistical parameters including central tendency (e.g. means) or other basic estimates (e.g. regression coefficient) AND variation (e.g. standard deviation) or associated estimates of uncertainty (e.g. confidence intervals)
☐	☒	For null hypothesis testing, the test statistic (e.g. *F*, *t*, *r*) with confidence intervals, effect sizes, degrees of freedom and *P* value noted<br>*Give P values as exact values whenever suitable.*
☒	☐	For Bayesian analysis, information on the choice of priors and Markov chain Monte Carlo settings
☒	☐	For hierarchical and complex designs, identification of the appropriate level for tests and full reporting of outcomes
☒	☐	Estimates of effect sizes (e.g. Cohen's *d*, Pearson's *r*), indicating how they were calculated

Our web collection on statistics for biologists contains articles on many of the points above.

Software and code

Policy information about availability of computer code

Data collection	No software was used for data collection.
Data analysis	Zen 3.0 (blue edition), MS Excel 2019, Adobe illustrator 2022, ExpressionSuite Software, GraphPad Prism 8.0 , ImageJ (V1.8.0), BD CellQuest Pro, Biacore T200, Enrichr (Panther 2016, PPI Hub proteins)

For manuscripts utilizing custom algorithms or software that are central to the research but not yet described in published literature, software must be made available to editors and reviewers. We strongly encourage code deposition in a community repository (e.g. GitHub). See the Nature Portfolio guidelines for submitting code & software for further information.

Data

Policy information about availability of data

All manuscripts must include a data availability statement. This statement should provide the following information, where applicable:

- Accession codes, unique identifiers, or web links for publicly available datasets
- A description of any restrictions on data availability
- For clinical datasets or third party data, please ensure that the statement adheres to our policy

All data generated or analyzed during this study are included in this published article (and its supplementary information files).

Human research participants

Policy information about studies involving human research participants and Sex and Gender in Research.

Reporting on sex and gender	We included IBD cohort from Severance Hospital which includes an inflamed biopsy dataset (CD and UC) and non-inflamed biopsy dataset (Control).<br>1) CD (inflamed biopsy dataset): mean age(at biopsy collection) 28.7, SD=7.1, 100% male<br>2) UC (inflamed biopsy dataset): mean age(at biopsy collection) 37.3, SD=11.3, 76.9% male, 23.1% female<br>2) Control (non-inflamed biopsy dataset): mean age(at biopsy collection) 64.7, SD=14.4, 60% male, 40% female
Population characteristics	Subjects age 18 to 89 with control and IBD patients (which included CD and UC diagnosis) were collected. All clinical data for subjects was current at the time of sample collection. Flare and remission classifications were defined by gastroenterologist assessment for standard of care.
Recruitment	Participants volunteer in sample collection. Informed consent forms were available for all participants and all were 18 years or older at the time of sample collection. There is no self-selection standard. All subjects were patients of Severance Hospital.
Ethics oversight	All participants signed an informed consent form prior sample collection. All samples were approved under provision of the Institutional Review Board of Severance Hospital.

Note that full information on the approval of the study protocol must also be provided in the manuscript.

Field-specific reporting

Please select the one below that is the best fit for your research. If you are not sure, read the appropriate sections before making your selection.

☒ Life sciences ☐ Behavioural & social sciences ☐ Ecological, evolutionary & environmental sciences

For a reference copy of the document with all sections, see nature.com/documents/nr-reporting-summary-flat.pdf

Life sciences study design

All studies must disclose on these points even when the disclosure is negative.

Sample size	Sample sizes are set to 3 or more based on the number of replicates necessary to achieve statistical significance derived from previous studies and our experiences (PMID: 36057640, PMID: 31604943, PMID: 28168305).
Data exclusions	No data were excluded from the analysis.
Replication	Results were consistently replicated across experiments as indicated in figure legends
Randomization	Mice were assigned randomly to experimental and control groups. Other experiments were not relevant as there were not grouping used in this study.
Blinding	This is an observational study and not clinical trial, thus blining is not applicable. Histopathological assessment of mice was performed blindly, by a single observer. The investigators were not blinded during sample collection. Blinding was used during tissue analysis.

Reporting for specific materials, systems and methods

We require information from authors about some types of materials, experimental systems and methods used in many studies. Here, indicate whether each material, system or method listed is relevant to your study. If you are not sure if a list item applies to your research, read the appropriate section before selecting a response.

Materials & experimental systems

n/a	Involved in the study	
☐	☒	Antibodies
☐	☒	Eukaryotic cell lines
☒	☐	Palaeontology and archaeology
☐	☒	Animals and other organisms
☒	☐	Clinical data
☒	☐	Dual use research of concern

Methods

n/a	Involved in the study	
☒	☐	ChIP-seq
☐	☒	Flow cytometry
☒	☐	MRI-based neuroimaging

Antibodies

Antibodies used

All antibodies used in the study have been listed in supplementray Data 2 (Manufacturer, catalog number).
Anti-ß-actin Santa Cruz Biotechnology sc-47778 WB (1:1000)
Anti-ß-catenin Santa Cruz Biotechnology sc-7963 WB (1:1000), IFA (1:200)
Anti-CD44 Santa Cruz Biotechnology sc-7297 WB (1:1000), IFA (1:200)
Anti-Cofilin1 (CFL1) Abcam ab11062 WB (1:2000)
Anti-E-cadherin Cell Signaling Technology #14472 WB (1:2000), IFA (1:200), Flow cytometry (1:100)
Anti-E-cadherin Proteintech #20874-1-AP IHC (1:1000~1:2000)
Anti-Ezrin/Radixin/Moesin (ERM) Cell Singaling Technology #3142 WB (1:500), IFA (1:100)
Anti-GAPDH ThermoFisher MA5-15738 WB (1:1000)
Anti-Moysin Light Chain 2 (MLC2) Cell Signaling Technology #3672 WB (1:300)
Anti-PAK1 Cell Signaling Technology #2602 WB (1:1000)
Anti-phospho-Cofilin1 (p-CFL1) (Ser3) Cell Signaling Technology #3313 WB (1:500)
Anti-phospho-Ezrin/Radixin/Moesin (ERM) Cell Singaling Technology #3726 WB (1:300), IFA (1:100)
Anti-phospho-Myosin Light Chain 2 (p-MLC2) (Thr18/Ser19) Cell Signaling Technology #3674 WB (1:300), IFA (1:100)
Anti-Phospho-LIMK1/2 (p-LIMK1/2) (Thr 508, 505) Cell Signaling Technology #3841 WB (1:1000)
Anti-Profilin 1 Cell Signaling Technology #3237 WB (1:2000)
Anti-RhoA Santa Cruz Biotechnology sc-418 WB (1:2000)
Anti-ROCK1 Abcam ab45171 WB (1:1000)
Anti-ROCK1 Cell Signaling Technology #4035 WB (1:1000), IP (1 μg)
Anti-TRIM40 Abclon AC181116-232 IHC (1:1000), WB (1:500)
Anti-Tubulin Applied Biological Materials G094 WB (1:3000)
Anti-Tubulin Santa Cruz Biotechnology sc-23948 WB (1:3000)
Anti-LIMK1 Cell Signaling Technology #3842 WB (1:1000)
Anti-Ubiquitin Santa Cruz Biotechnology sc-8017 WB (1:2000)
Anti-Vinculin Santa Cruz Biotechnology sc-73614 WB (1:2000), IFA (1:200)
Anti-FLAG Novus Biologicals NBP1-06712 WB (1:1000)
Anti-FLAG Applied Biological Materials G191 WB (1:1000), IFA (1:200)
Anti-Myc Cell Signaling Technology #2276 WB (1:1000)
Anti-Myc Cell Signaling Technology #2278 IFA (1:100-1:200)
Anti-GFP Roche #11814460001 IP (0.6 μg)
Anti-GFP Santa Cruz Biotechnology sc-9996 WB (1:2000)
Alexa Fluor 568-conjugated Phalloidin ThermoFisher A12380 IFA (1:300)
Alexa Fluor 594-conjugated DNase I ThermoFisher D12373 IFA (1:100~1:200)
Alexa Fluor 488-conjugated secondary mouse antibodies ThermoFisher #A-11029 IFA (1:300)
Alexa Fluor 488-conjugated secondary rabbit antibodies ThermoFisher # A-11034 IFA (1:300)
Alexa Fluor 568-conjugated secondary mouse antibodies ThermoFisher #A-11031 IFA (1:300)
Alexa Fluor 568-conjugated secondary rabbit antibodies ThermoFisher #A-11036 IFA (1:300)
Alexa Fluor 647-conjugated secondary antibody ThermoFisher #A-21245 IFA (1:300)
goat anti-mouse HRP ThermoFisher #31430 WB (1:5000~1:10000)
goat anti-rabbit HRP ThermoFisher #31460 WB (1:5000~1:10000)
goat anti-rat HRP Bethyl Laboratories #A110-305P WB (1:10000)

Validation

All primary antibodies were confirmed on the species and application through the validation statement on the manufacturer's website and their use in the literature.
Mouse anti-ß-actin Santa Cruz Biotechnology - validated in 10,658 publications referenced
Mouse anti-ß-catenin Santa Cruz Biotechnology - validated in 791 publications referenced
Mouse anti-CD44 Santa Cruz Biotechnology - validated in 126 publications referenced
Rabbit anti-Cofilin1 (CFL1) Abcam - validated in 30 publications referenced
Mouse anti-E-cadherin Cell Signaling Technology - validated in 686 publications referenced
Rabbit anti-E-cadherin Proteintech - validated in 1,292 publications referenced
Rabbit anti-Ezrin/Radixin/Moesin (ERM) Cell Singaling Technology- validated in 89 publications referenced
Mouse anti-GAPDH ThermoFisher - validated in 653 publications referenced
Rabbit anti-Moysin Light Chain 2 (MLC2) Cell Singaling Technology - validated in 299 publications referenced
Rabbit anti-PAK1 Cell Signaling Technology - validated in 196 publications referenced
Rabbit anti-phospho-Cofilin1 (p-CFL1) (Ser3) - validated in 227publications referenced
Rabbit anti-phospho-Ezrin/Radixin/Moesin (ERM) Cell Singaling Technology - validated in 75publications referenced
Rabbit anti-phospho-Myosin Light Chain 2 (p-MLC2) (Thr18/Ser19) Cell Signaling Technology- validated in 386 publications referenced
Rabbit anti-Phospho-LIMK1/2 (p-LIMK1/2) (Thr 508, 505) Cell Signaling Technology - validated in 85 publications referenced
Rabbit anti-Profilin 1 Cell Signaling Technology - validated in 30 publications referenced
Mouse anti-RhoA Santa Cruz Biotechnology - validated in 1,179 publications referenced
Rabbit anti-ROCK1 Abcam - validated in 126 publications referenced
Rabbit anti-ROCK1 Cell Signaling Technology - validated in 129 publications referenced
Rabbit anti-TRIM40 was validated for use in IHC and WB by our laboratory
Mouse anti-Tubulin Applied Biological Materials validated in 5 publications referenced
Mouse anti-Tubulin Santa Cruz Biotechnology - validated in 441 publications referenced
Rabbit anti-LIMK1 Cell Signaling Technology - validated in 85 publications referenced
Mouse anti-Ubiquitin Santa Cruz Biotechnology - validated in 2,606 publications referenced
Mouse anti-Vinculin Santa Cruz Biotechnology - validated in 258 publications referenced
Rat anti-FLAG Novus- validated in 80 publications referenced
Mouse anti-FLAG Applied Biological Materials - validated in 4 publications referenced
Mouse anti-Myc Cell Signaling Technology - validated in 1,687 publications referenced
Rabbit anti-Myc Cell Signaling Technology - validated in 584 publications referenced

Mouse anti-GFP Roche - validated in 1,987 publications referenced
Mouse anti-GFP Santa Cruz Biotechnology - validated in 2,666 publications referenced

Eukaryotic cell lines

Policy information about cell lines and Sex and Gender in Research

Cell line source(s)	HT-29 (HTB-38, ATCC), HCT 116 (CCL-247, ATCC), U937 (CRL-1593.2, ATCC), Caco-2 (HTB-37, ATCC), HEK 293T (CRL-11268, ATCC), 293A (R70507, Invitrogen), HeLa (CCL-2, ATCC), A549 (CCL-185, ATCC), MDA-MB-231 (CRM-HTB-26, ATCC),FHC (CRL-1831, ATCC)
Authentication	All the cell lines were authenticated by ATCC and Invitrogen. STR profiling was used for authentication by ATCC and Invitrogen.
Mycoplasma contamination	Cell lines tested negative for mycoplasma.
Commonly misidentified lines (See ICLAC register)	No commonly misidentified cell lines were used in the study.

Animals and other research organisms

Policy information about studies involving animals; ARRIVE guidelines recommended for reporting animal research, and Sex and Gender in Research

Laboratory animals	C57BL/6N (4–8 weeks, OrientBio) and Trim40-/- mice were maintained in the specific pathogen-free facility according to Korean Food and Drug Administration guidelines. The housing conditions are as follows. Light cycle: A 14-hour light/10-hour dark cycle. Temperature and humidity: 18-23C with 40-60% humidity.
Wild animals	No wild animals were used in the study.
Reporting on sex	Since male mice are more susceptible to colitis than female mice, male mice were used for the colitis models.
Field-collected samples	No field collected samples were used in the study.
Ethics oversight	All animal experiments were reviewed and approved by the Institutional Animal Care and Use Committee of the Yonsei University.

Note that full information on the approval of the study protocol must also be provided in the manuscript.

Flow Cytometry

Plots

Confirm that:

☒ The axis labels state the marker and fluorochrome used (e.g. CD4-FITC).

☒ The axis scales are clearly visible. Include numbers along axes only for bottom left plot of group (a 'group' is an analysis of identical markers).

☒ All plots are contour plots with outliers or pseudocolor plots.

☒ A numerical value for number of cells or percentage (with statistics) is provided.

Methodology

Sample preparation	All samples were prepared in a single-cell suspension form using cell line.
Instrument	FACScalibur
Software	BD CellQuest Pro
Cell population abundance	> 10,000 cells were analyzed
Gating strategy	FSC/SSC gating to exclude debris. No other gating was applied.

☒ Tick this box to confirm that a figure exemplifying the gating strategy is provided in the Supplementary Information.
