# Supplementary material for: TRIM40 is a pathogenic driver of inflammatory bowel disease subverting intestinal barrier integrity
Source: Nat Commun. 2023 Feb 9;14:700. doi: 10.1038/s41467-023-36424-0 (PMC9908899; doi:10.1038/s41467-023-36424-0)

Uncropped images of agarose gels and immnunoblots

Fig. 2c

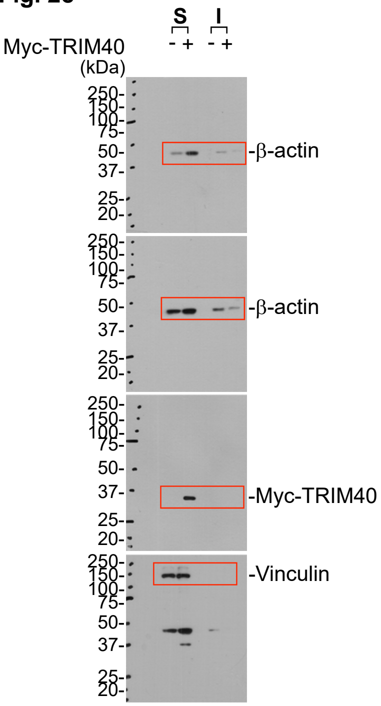

Fig. 2d

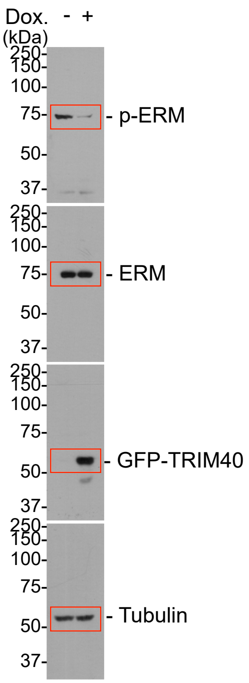

Fig. 2f

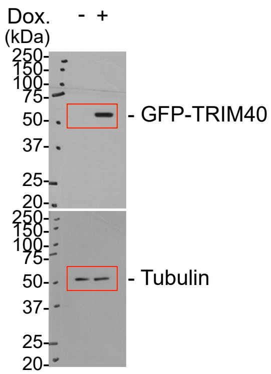

Fig. 3b

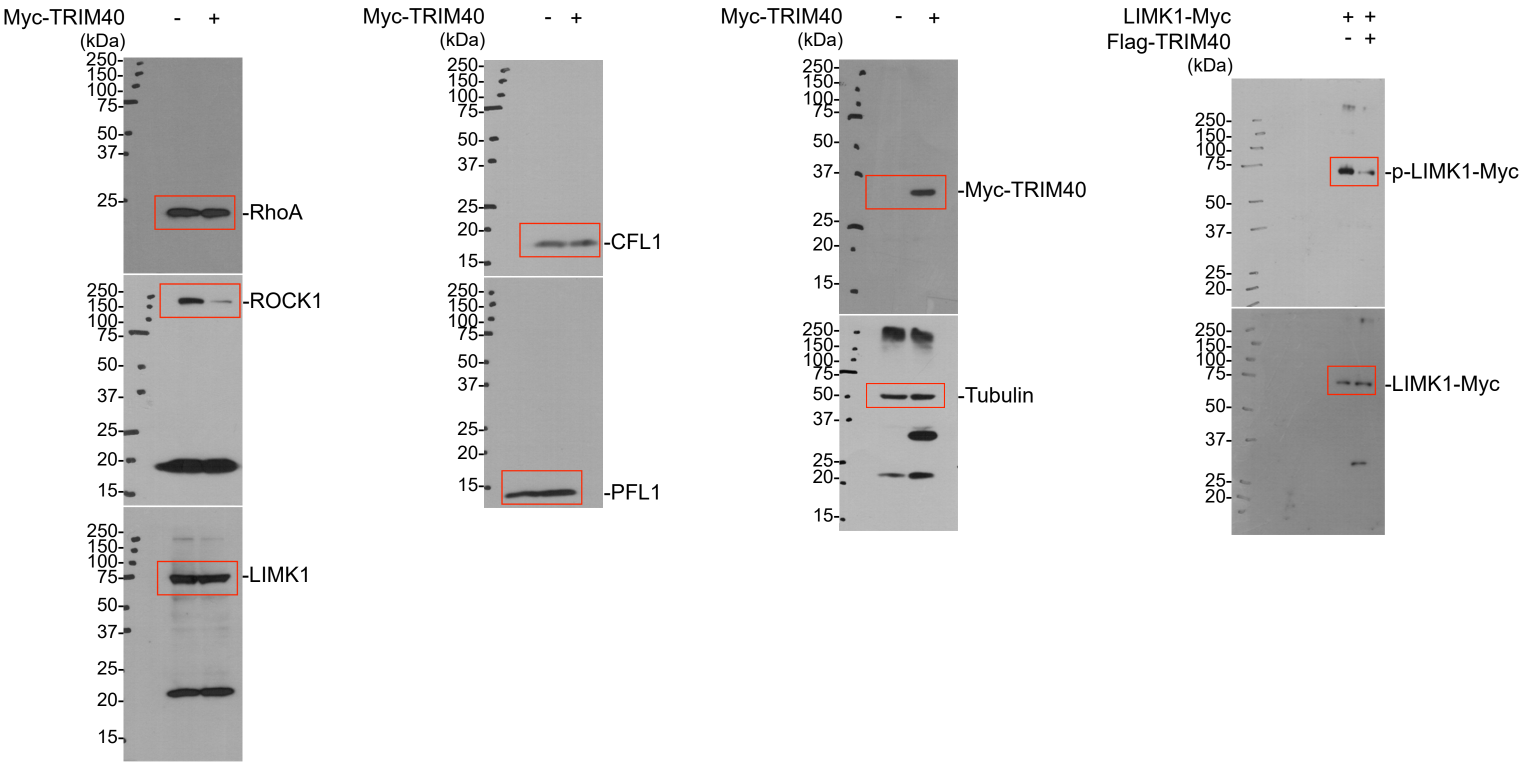

Uncropped images of agarose gels and immnunoblots (Continuation)

Fig. 3b (Continuation)

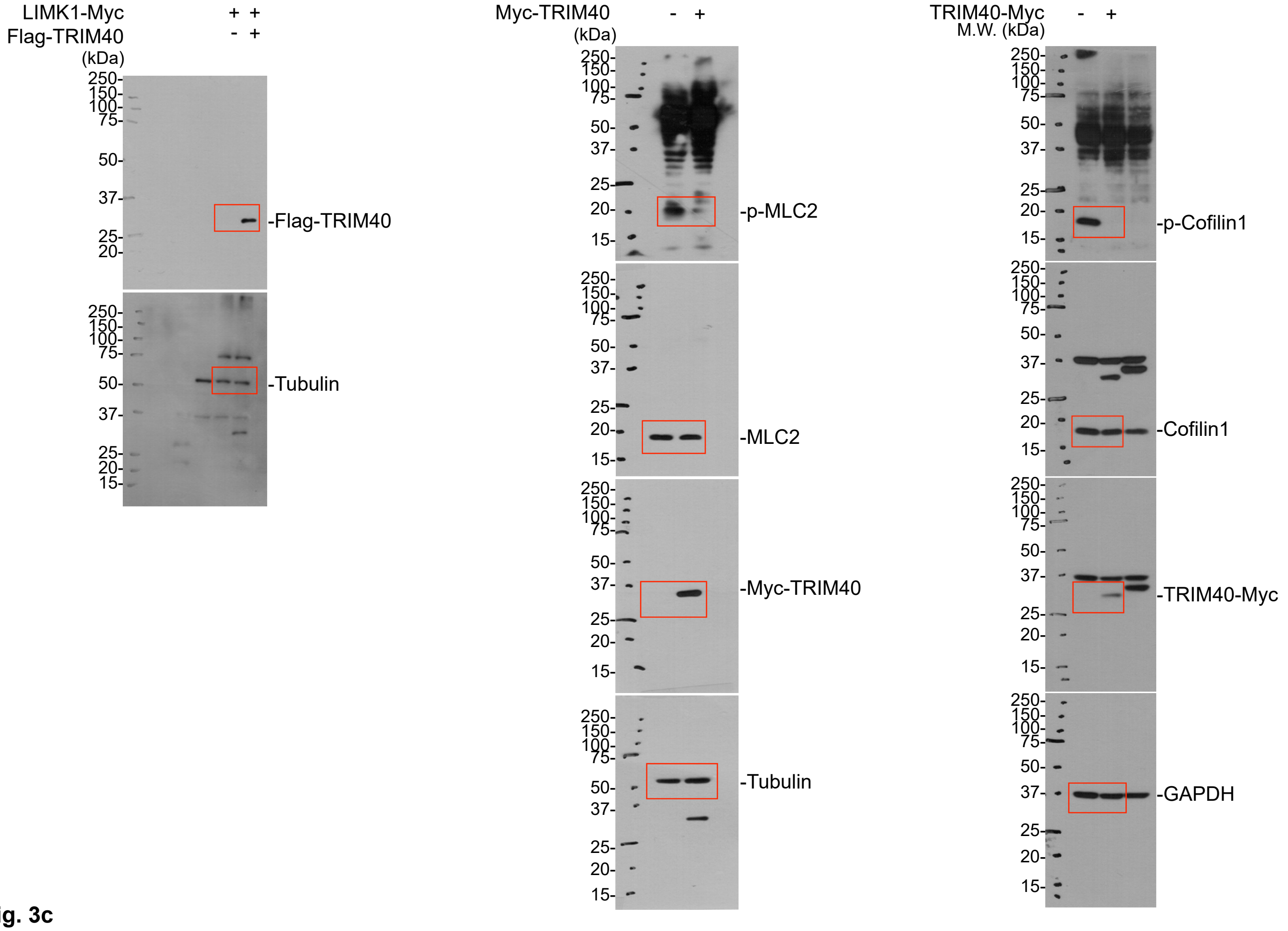

Fig. 3c

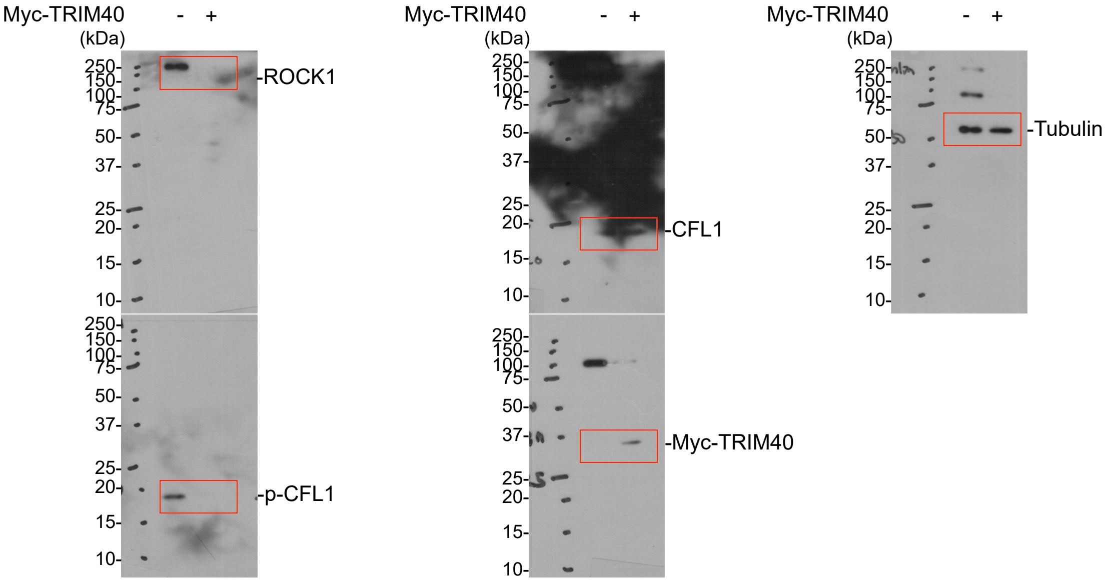

Uncropped images of agarose gels and immnunoblots (Continuation)

Fig. 3d

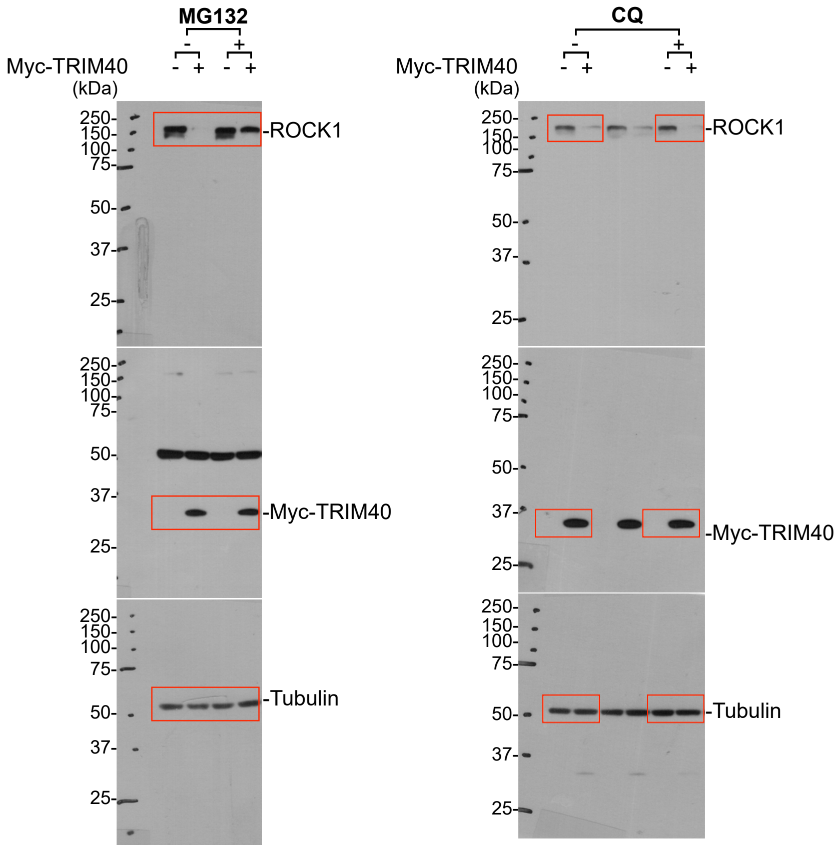

Fig. 3e

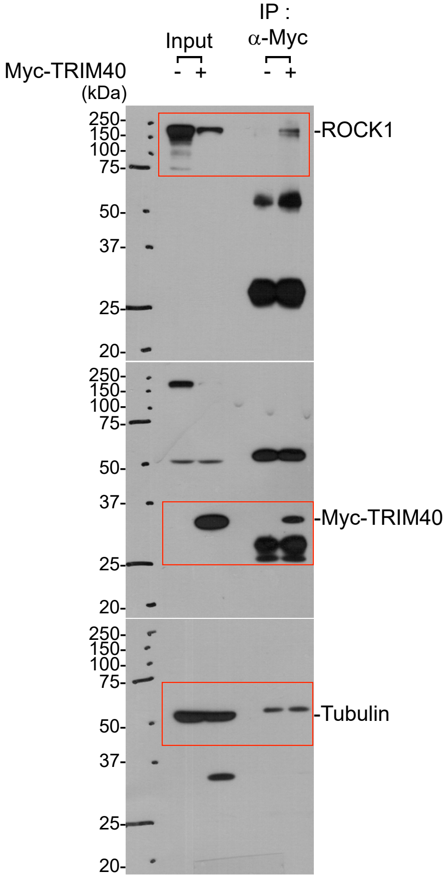

Fig. 3f

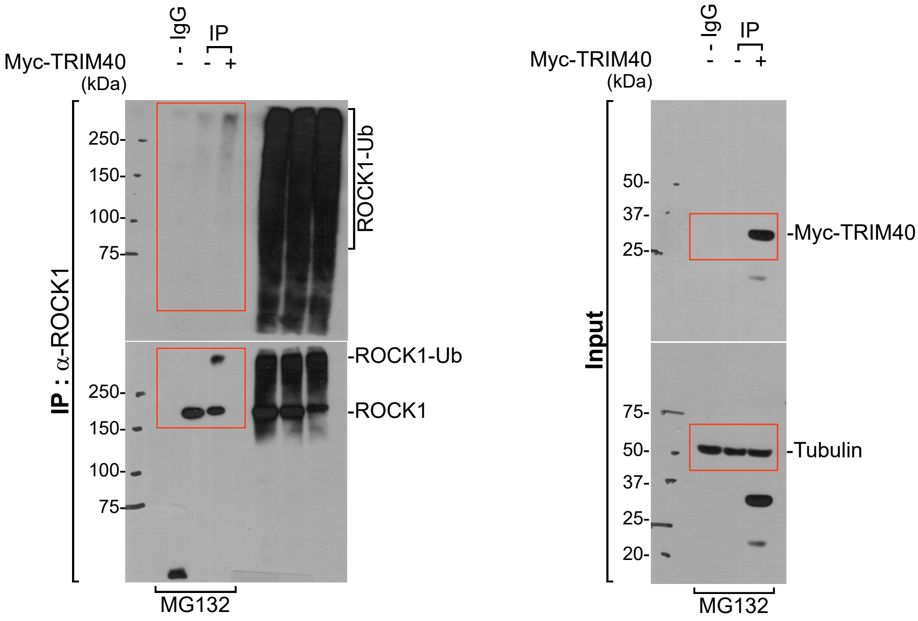

Uncropped images of agarose gels and immnunoblots (Continuation)

Fig. 4b

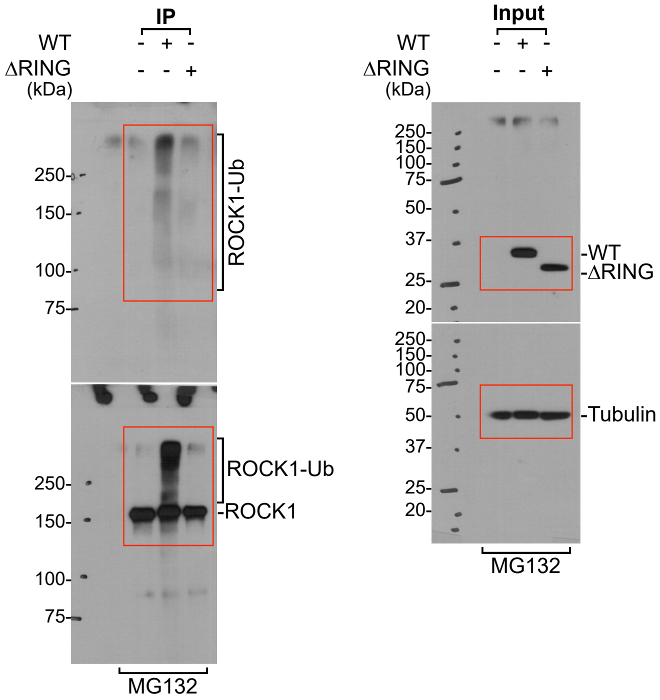

Fig. 4c

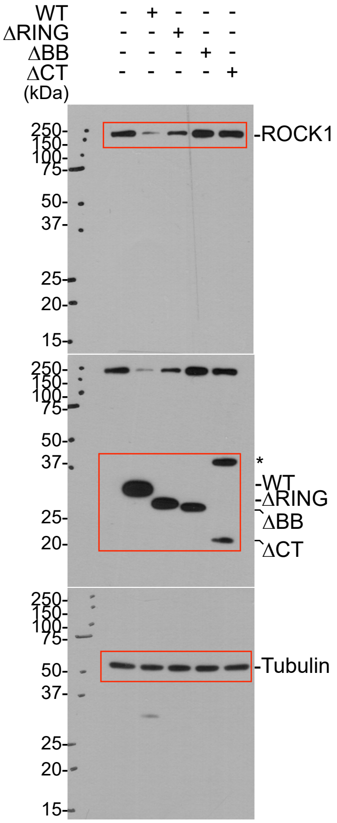

Fig. 4d

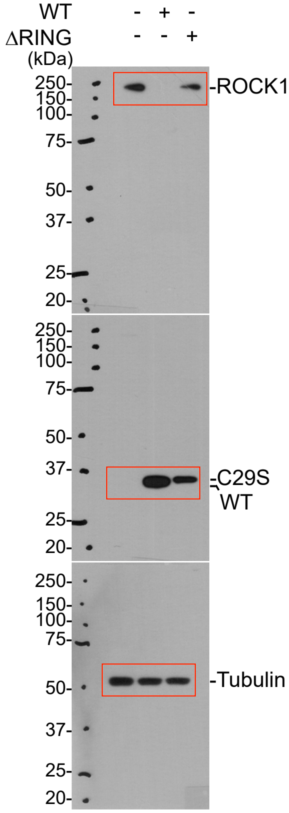

Fig. 4f

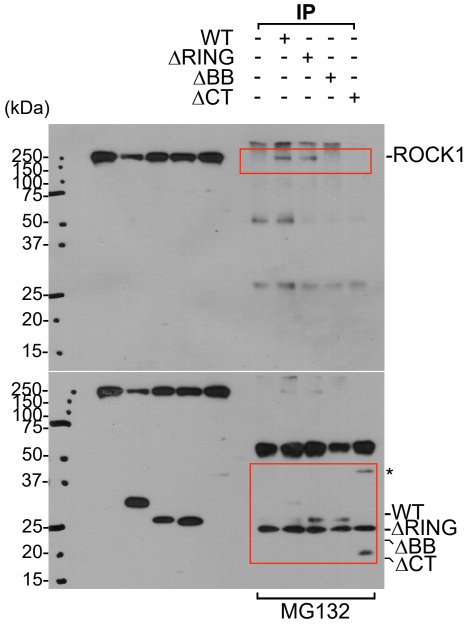

Fig. 5h

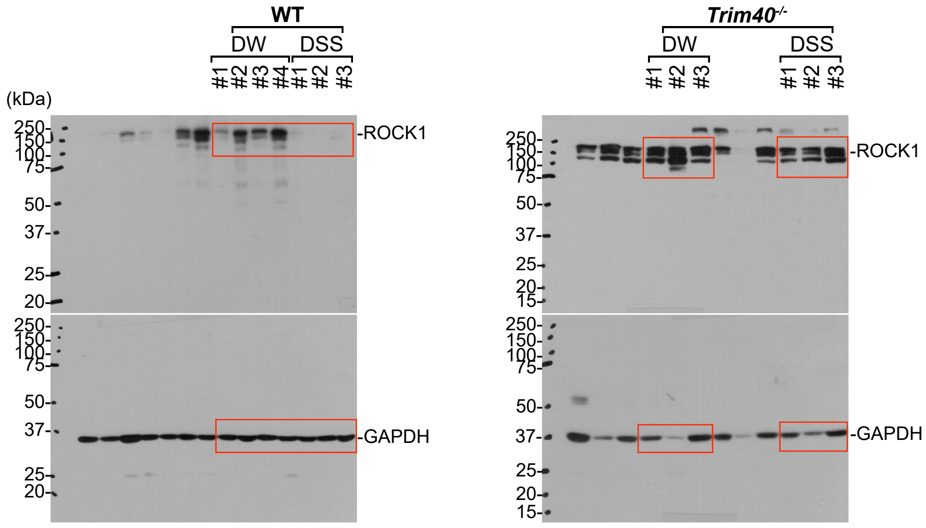

Uncropped images of agarose gels and immnunoblots (Continuation)

Supplementary Fig. 2b

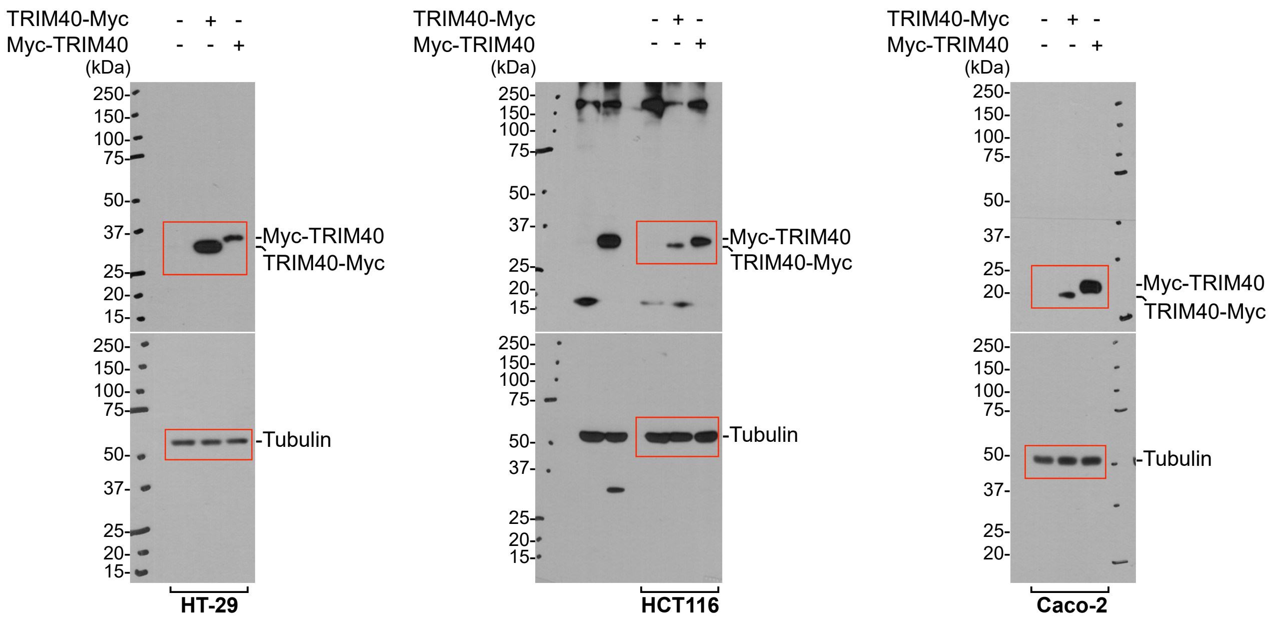

Supplementary Fig. 3e

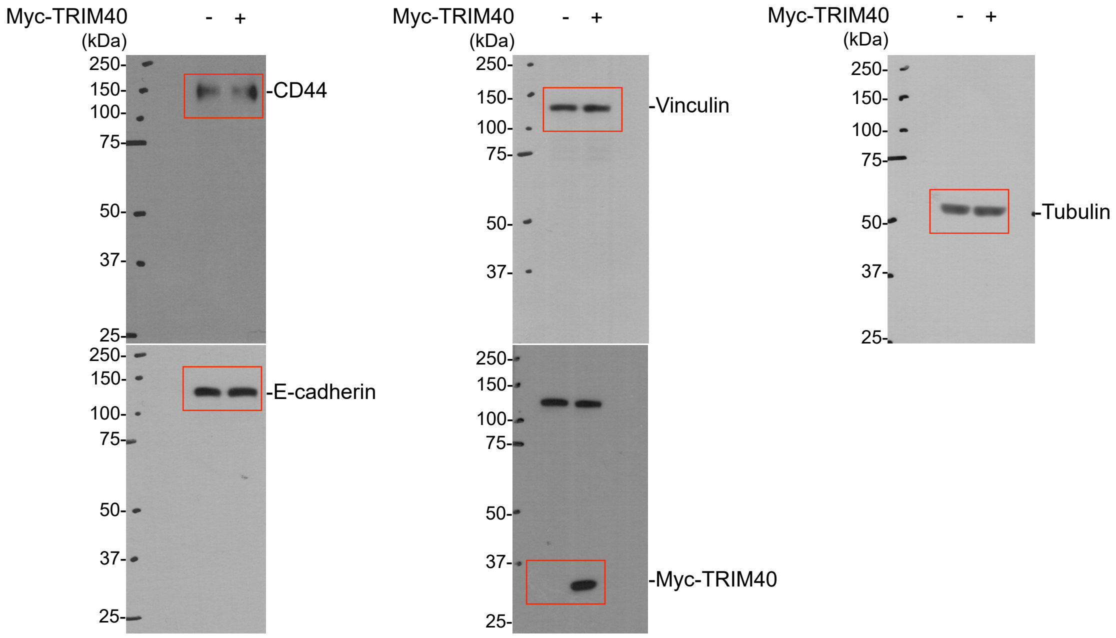

Supplementary Fig. 3g

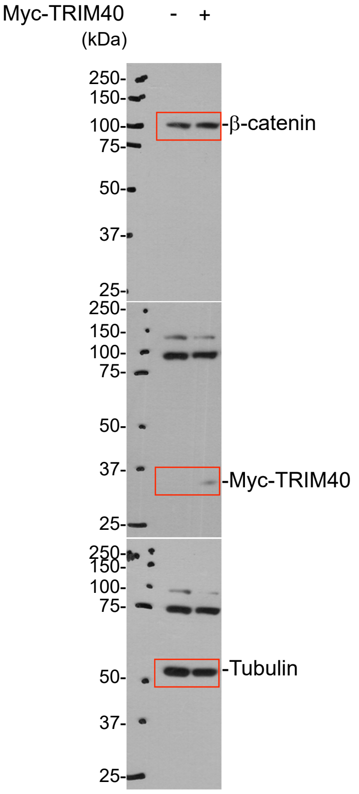

Uncropped images of agarose gels and immnunoblots (Continuation)

Supplementary Fig. 4a

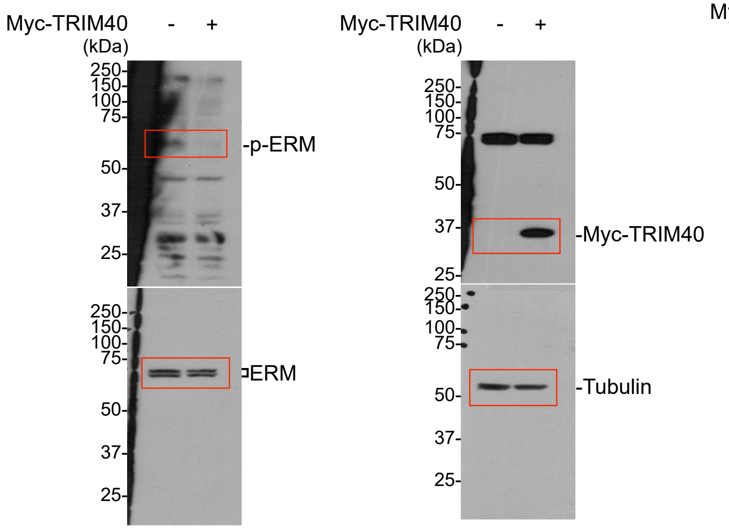

Supplementary Fig. 4c

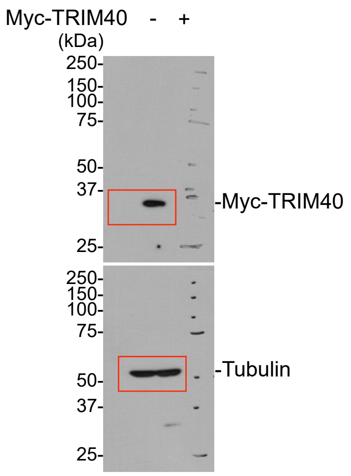

Supplementary Fig. 4d

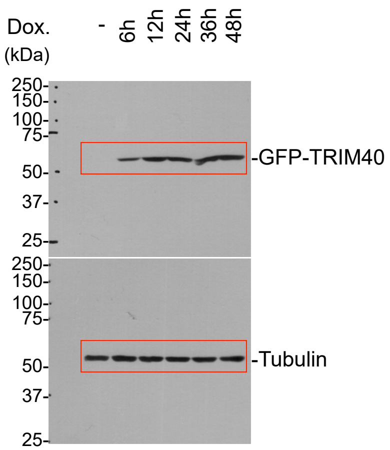

Supplementary Fig. 5c

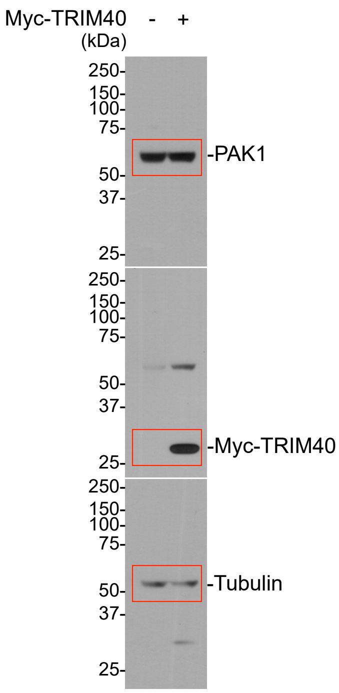

Supplementary Fig. 5d

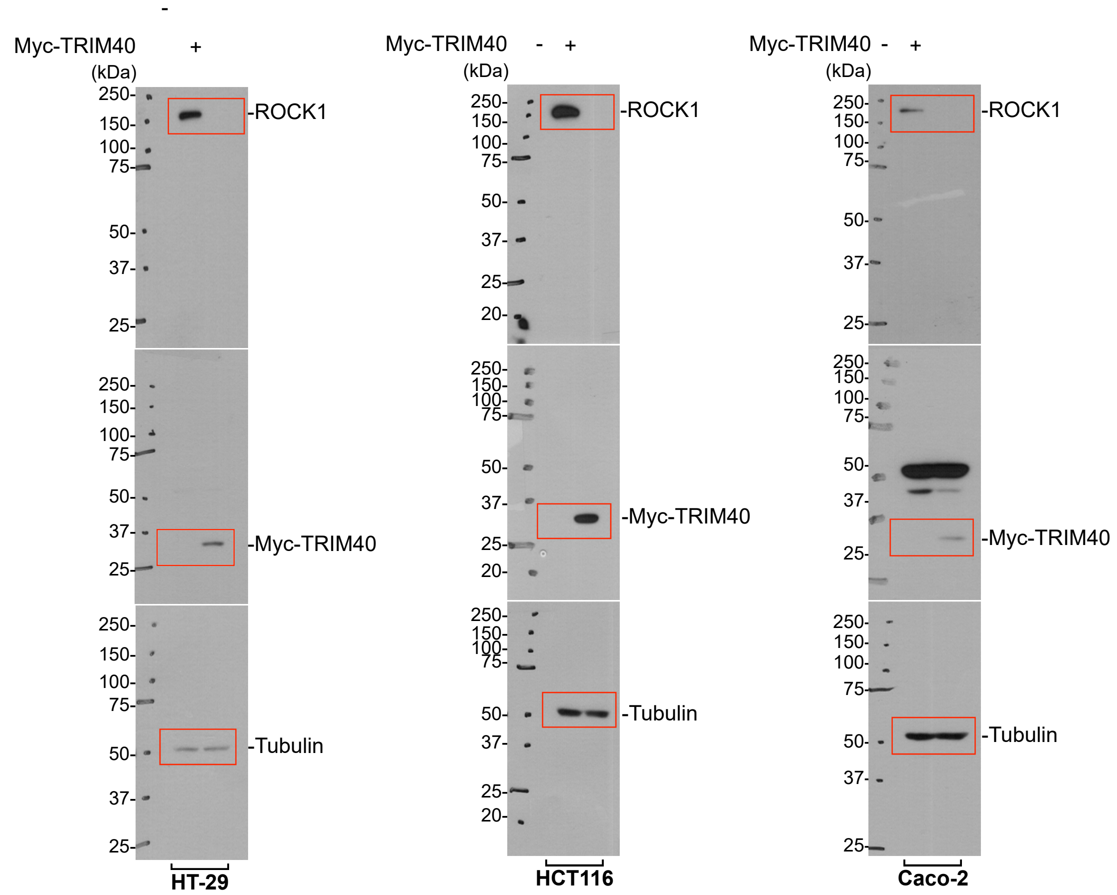

Uncropped images of agarose gels and immnunoblots (Continuation)

Supplementary Fig. 5e

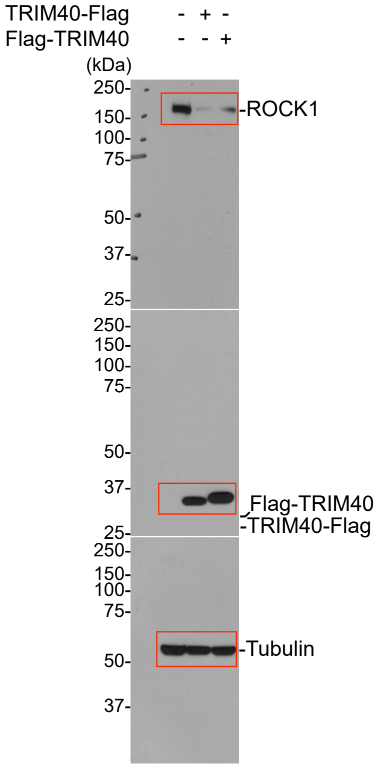

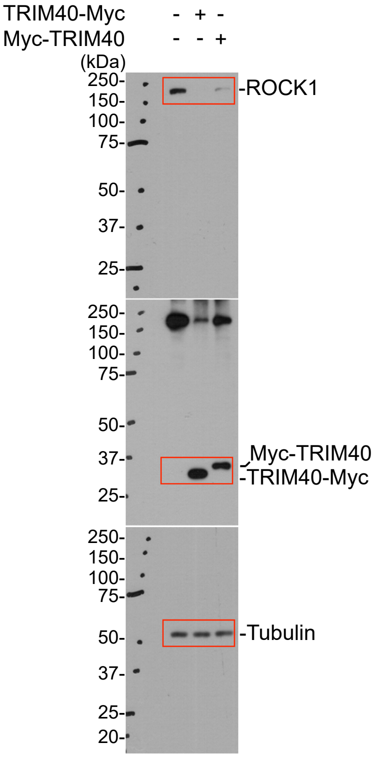

Supplementary Fig. 5g

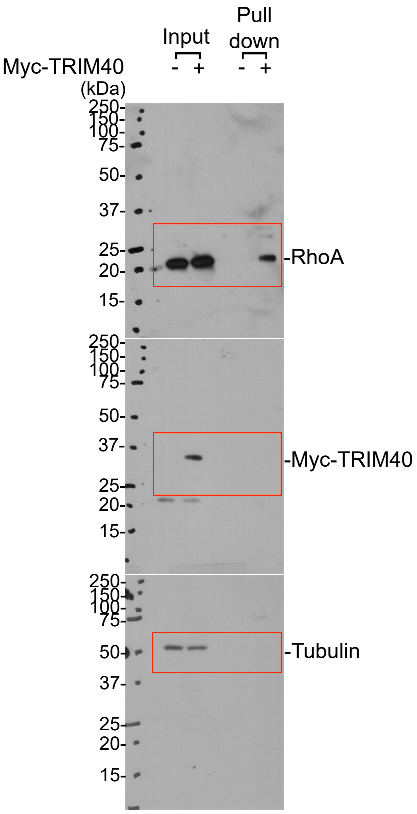

Supplementary Fig. 6

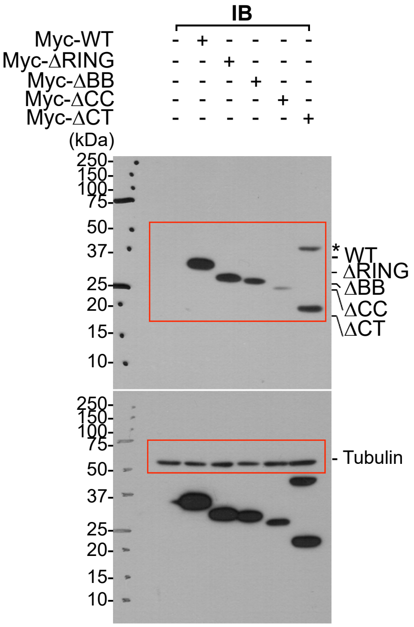

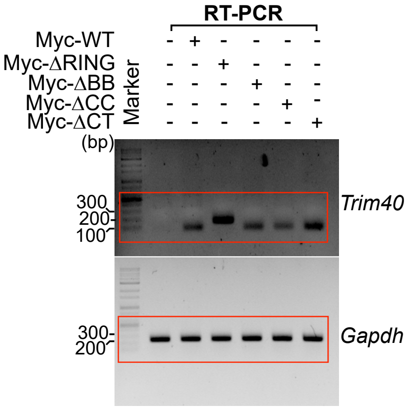

Supplementary Fig. 8a

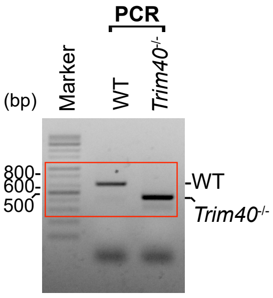

Supplement: Supplementary file 7 — Source Data [file 41467_2023_36424_MOESM7_ESM.zip › Source data for uncropped gels.pdf]
